# Rare Case of Cystic Lymphangioma Transforming Into Lymphangiosarcoma: A Case Report

**DOI:** 10.3389/fonc.2022.814023

**Published:** 2022-02-18

**Authors:** Huili Yu, Qinxiang Mao, Lingyan Zhou, Jiawei Li, Xunhua Xu

**Affiliations:** ^1^ Department of Radiology, China Resources & WISCO General Hospital, Wuhan University of Science and Technology, Wuhan, China; ^2^ Department of Radiology, Liuzhou People’s Hospital, Liuzhou, China; ^3^ Department of Radiology, The Second Hospital of Wuhan Iron and Steel Company, Wuhan, China

**Keywords:** cystic lymphangioma, lymphangioma, lymphangiosarcoma, malignant transformation, case report

## Abstract

Cystic lymphangioma (CL) is a rare benign tumor that mainly occurs in the neck and axilla and usually occurs in children, whereas lymphangiosarcoma (LAS) is a rare invasive tumor, usually secondary to chronic lymphedema caused by various causes, with a rare malignant transformation from CL. We presented the case of a 63-year-old woman who underwent four surgical excisions for multiple recurrence of CL in the right groin. The changes of imaging and pathological examination revealed the unusual process of its gradual malignant transformation into LAS. We followed up the patient for 16 years, and she eventually died of LAS complications.

## Introduction

Lymphangioma (LA) is a rare benign tumor. The common clinical type of LA is cystic lymphangioma (CL) (1), and surgical resection is its main treatment. Lymphangiosarcoma (LAS) is a rare malignant tumor that is almost always secondary to chronic edema caused by various causes (mainly after surgery or radiotherapy) (2, 3). Imaging is the main means of examination before surgery (4) and shows multilocular cystic lesions with uniform signal/density, smooth cyst wall, and thin septum (5, 6), whereas LAS shows soft tissue components in the cyst (3). The proliferation of endothelial cells and nuclear atypia and division are observed under a microscope, and positive expression of lymphatic endothelial cells (D2-40) by immunohistochemistry is observed (7). To the best of our knowledge, the case of malignant transformation from CL to LAS has rarely been reported in the literature to date. This study presents a rare case of malignant transformation from CL to LAS after multiple surgical resections and recurrences and analyzes this rare evolution from clinical, imaging, and pathological perspectives.

## Case Presentation

**Figure 1:**
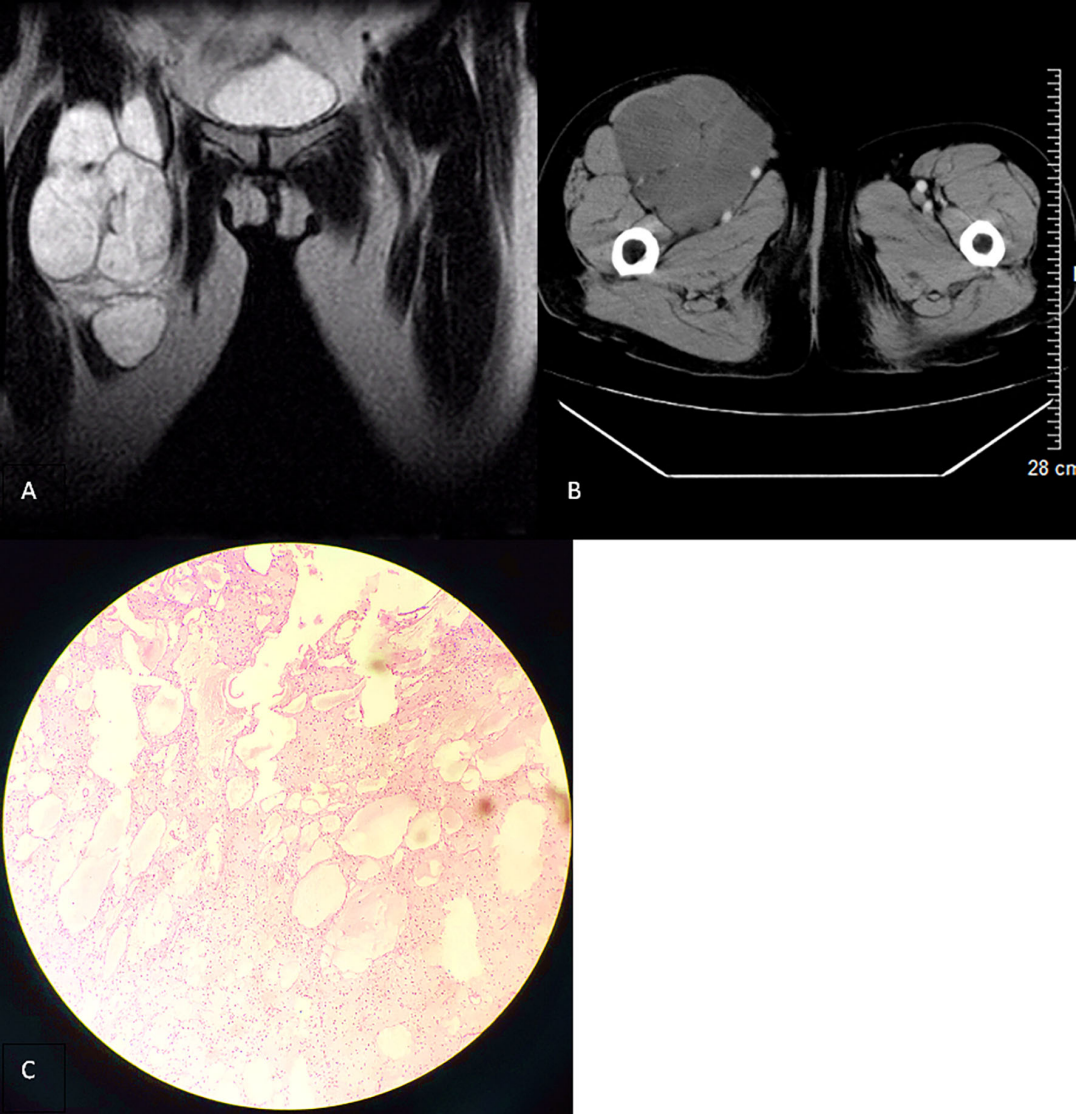
**(A)** T2-weighted imaging in the coronal section (2008) revealing a polycystic lesion in the right inguinal region with smooth border and low-signal intensity septum. **(B)** Contrast-enhanced computed tomography scan in the transverse section of the lesion with no significant enhancement. **(C)** Microscopically, there are several unequal thin-walled cavities, the inner wall is lined with monolayer flat epithelial cells, the cavity is full of protein fluid, the endothelial cells are loose, the nucleus is small, and no atypia is found (hematoxylin and eosin stain, ×100).

**Figure 2:**
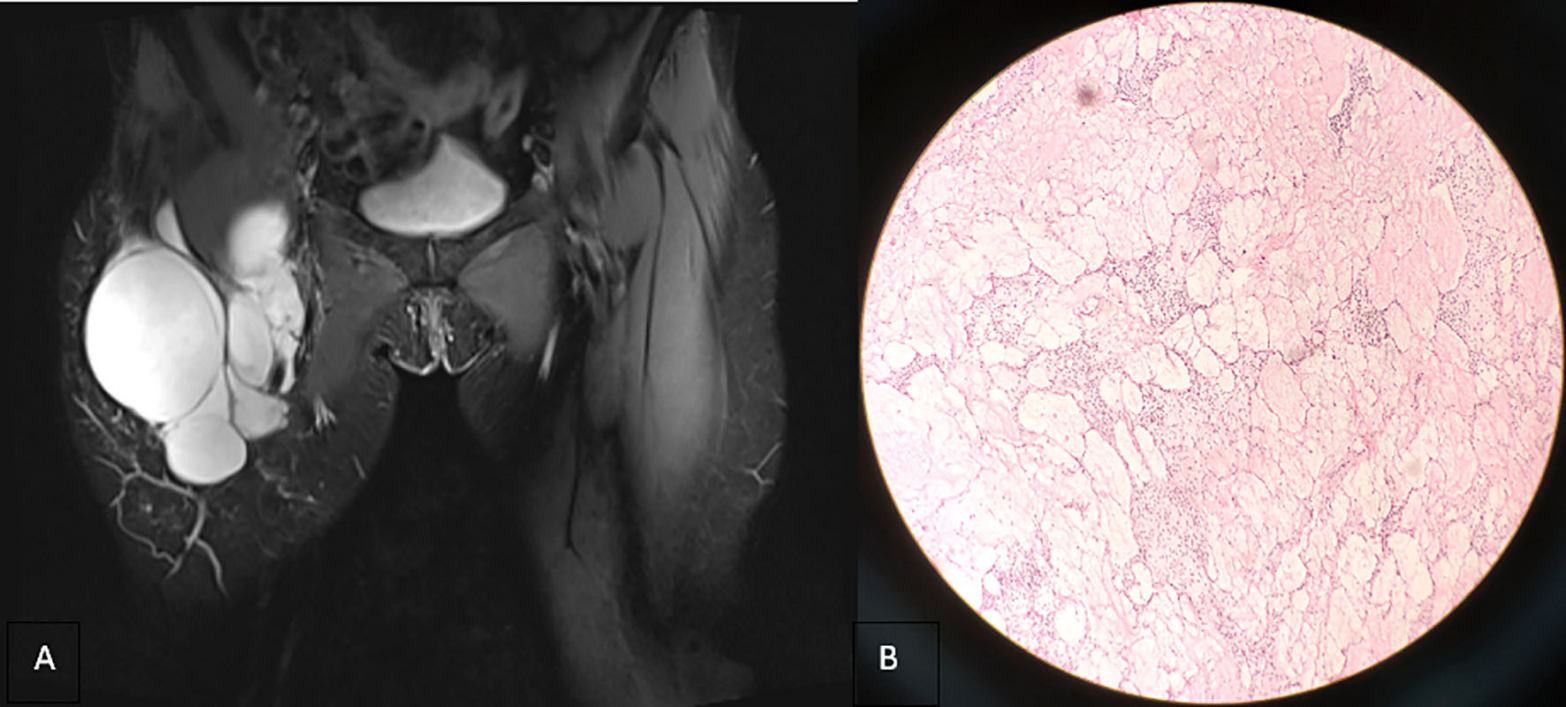
**(A)** Coronal T2-weighted imaging (2013) revealing that the lesion is still confined to the right inguinal area with uniform signal intensity and smooth cyst wall. **(B)** Microscopically, a number of dilated lymphatic vessels of different sizes are observed, the endothelial cells are slightly dense, the size of the nucleus is slightly different, and atypical cells are observed (hematoxylin and eosin stain, ×100).

A 63-year-old woman was hospitalized for the fourth recurrence of a right inguinal tumor and abdominal distension. The patient underwent the first surgical resection of the right inguinal mass in 2002 and was diagnosed with CL by pathological examination. Subsequently, the patient was hospitalized in 2008 and 2013 because of tumor recurrence. Preoperative imaging examination showed right inguinal polycystic lesions with uniform signal/density, low signal intensity on T1-weighted imaging, high signal intensity on T2-weighted imaging, and slender septum (**Figures 1** and **2**). No enhancement was observed on the computed tomography enhanced scan (**Figure 1B**). During the operation, the cystic mass confined to the right inguinal area showed multiple growths involving the femoral artery and vein, and the patient was subsequently diagnosed with CL in two pathological examinations (**Figures 1C** and **2B**), but atypical endothelial cells were observed in pathological sections in 2013 (**Figure 2B**).

**Figure 3:**
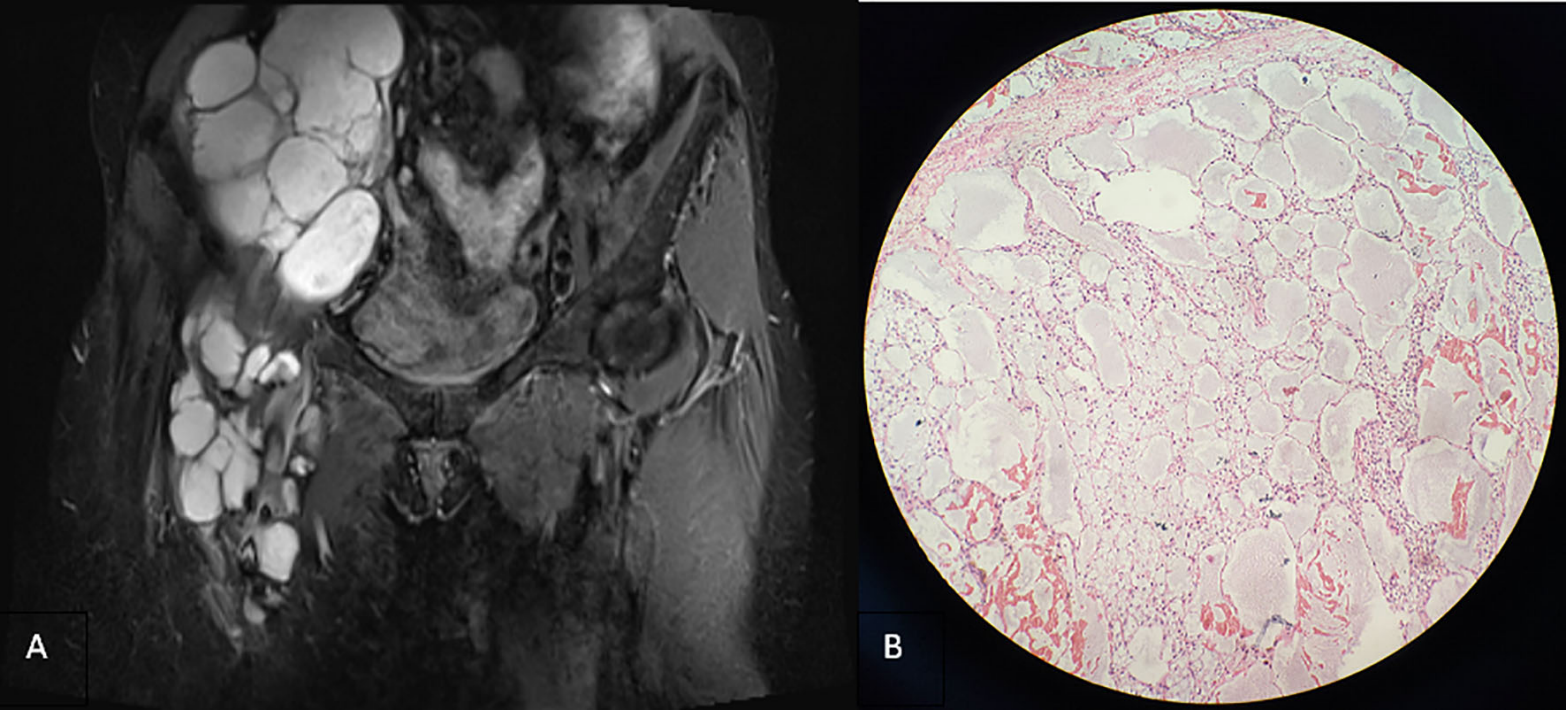
**(A)** Coronal T2-weighted imaging lipid pressure sequence (2015) revealing multiple cystic lesions of varying sizes in the right inguinal region, infiltrating and growing along the tissue space behind the peritoneum and the space between the anterior femoris muscle group, with uneven signal intensity, scattered spots and slightly low signal intensity, and less uniform thickness of the septum. **(B)** Microscopically, multiple cystic dilated lymphatic vessels of different sizes, partial hyperplasia, endothelial cell proliferation and accumulation, atypical nucleus, thick nuclear membrane, and polar disappearance of nucleus and nucleolus are observed in some cells (hematoxylin and eosin stain, ×200).

In 2016, the patient was hospitalized for surgical resection because of the third recurrence of the tumor. On the right side of the groin area, the children’s skull size irregular mass was palpable. It extended to the right lower abdomen, with soft texture, unclear boundary, no tenderness, and poor activity, which did not include the abdomen when lying down. Moreover, the patient’s lower limbs were not swollen. Magnetic resonance imaging (MRI) revealed that the right inguinal cystic lesion extended upward to the retroperitoneum with uneven signal intensity and thickened low-signal intensity septum (**Figure 3A**). During the operation, the 10 × 8 × 6-cm cystic was observed, and solid lobulated mass in the right inguinal region grew from the right femoral canal. The capsule was intact and densely adhered to the right femoral artery and vein, which was resected by sharp separation. Multiple cystic solid masses in the middle and upper segments of the right thigh were observed downward through the incision, with a maximum size of approximately 12 × 8 × 6 cm, which adhered to the right femur. Moreover, the mass was resected completely after careful separation. The right retroperitoneal cystic solid mass measuring 25 × 20 × 8 cm was observed upward through the incision. The right ureter and iliac vessels were exposed and protected, and the mass was completely resected. Gross specimen: three piles of gray white and gray red irregular tissues, showing translucent and mucilaginous, with gray red section, some of which were gray white and light yellow. Microscopic examination showed multiple cystic lymphatic vessels of different sizes surrounded by large stellate cells, with atypical nuclei, mitosis, necrosis, hemorrhage, and lymphatic endothelial cell proliferation, tending to LAS (**Figure 3B**). Immunohistochemical staining revealed the following: D2-40 (+), S-100 (±), cluster of differentiation 34 (−), smooth muscle actin (−), desmin (−), and cytokeratin 68 (−).

**Figure 4:**
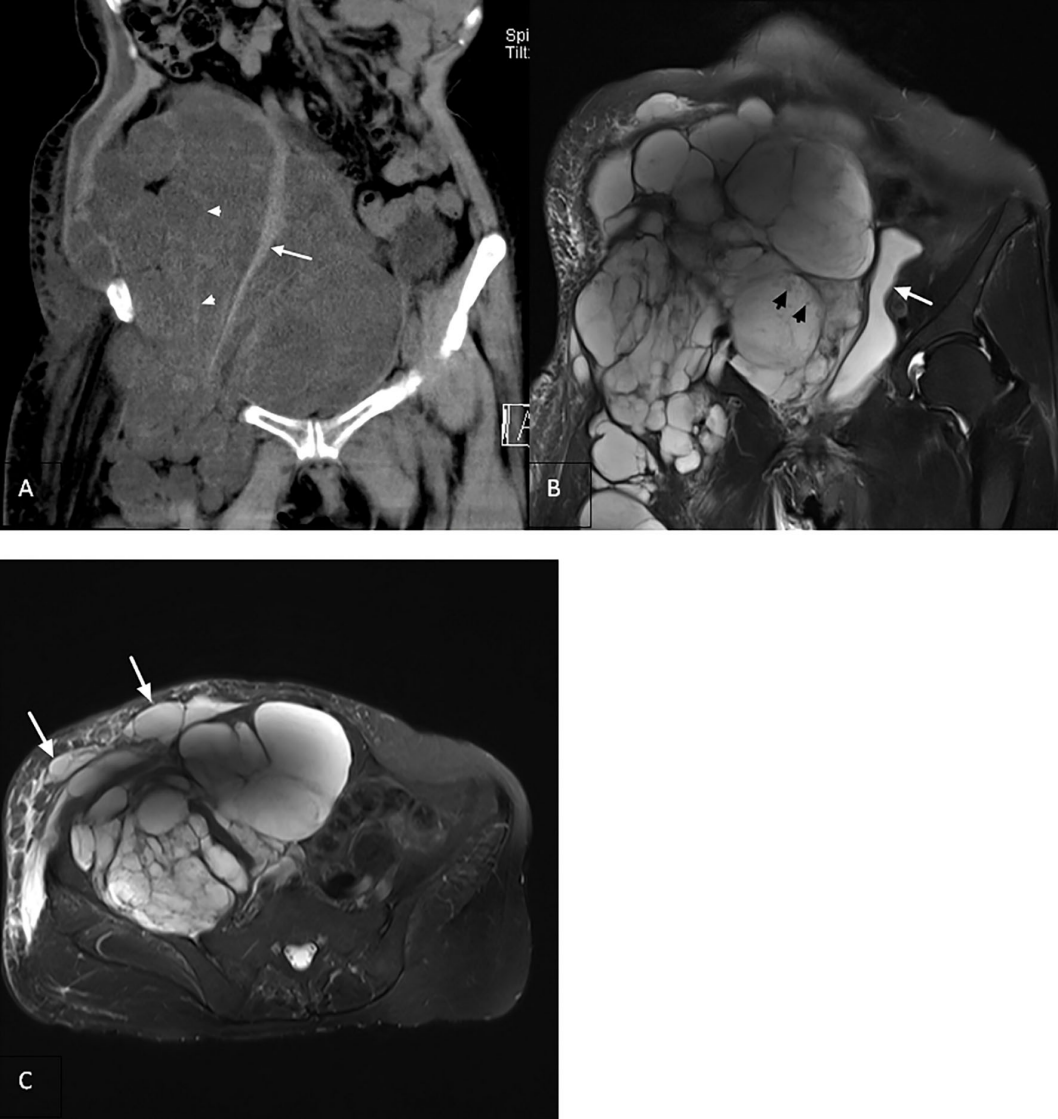
**(A)** Coronal computed tomography reconstruction (2018) showing a multilocular cystic solid heterogeneous mass in the retroperitoneum with poorly delineated boundaries (arrowhead). Right psoas major muscle showing compressed atrophy and decrease in volume (arrow). **(B)** Coronal T2-weighted imaging revealing that the lesion invaded the retroperitoneum, inguinal region, and upper right thigh with uneven signal intensity. There are many divergent spots of slightly lower signal intensity (arrowhead) in the lesion, with uneven thickness of the septum. The bladder is compressed and moved to the left (arrow). **(C)** Axial view showing the right abdominal cyst with lymphedema (arrow).

It had been 16 years since the patient was hospitalized in 2002 for the first inguinal tumor operation, and malignant transformation had been found for 23 months. She had high blood pressure in the past, did not receive treatment, underwent hysterectomy in 2003, and was allergic to iodophor. She denied a history of coronary heart disease, diabetes, and trauma. A large irregular mass extending to the right lower abdomen and right thigh was palpated in the right inguinal region. The right lower abdomen was evidently raised and asymmetrical with the left lower abdomen, with unclear boundaries, no tenderness, poor movement, and moderate edema of the right lower extremities. MRI revealed that the lesion involved the right retroperitoneum, pelvis, abdominal wall, groin, and muscle space of the middle and upper thigh on the right, surrounding or pushing the adjacent vessels. The signal/density in the capsule was uneven, and the septum was uneven or blurred (**Figures 4A, B**). Lymphedema and cystic dilated lymphatic vessels were observed in the right abdominal wall (**Figure 4C**). No other organ metastases were observed. Carbohydrate antigens, alpha-fetoprotein, and carcinoembryonic antigen levels were within the normal range. According to the surgeons, the scope of the tumor was significantly large and easy to relapse; therefore, conservative treatment was recommended. The patient was generally in poor condition and could not tolerate the response to chemotherapy; thus, she was provided targeted treatment. After 38 days of oral apatinib administration, the circumference of the root of the right thigh was reduced from 65 cm to 57 cm, but the patient’s blood pressure was unstable (up to 180/125 mmHg), and the antihypertensive effect of oral valsartan was not good. Moreover, albuminuria was observed simultaneously; therefore, apatinib was discontinued, and symptomatic treatment was performed. Due to the rapid growth of the tumor, compression of abdominal organs (double kidneys, ureters, colorectum) led to hydronephrosis and defecation disorders. Moreover, long-term bed rest led to pendant pneumonia and subsequently death after gradual failure of the heart, lungs, and other organs.

## Discussion

LA is a rare lymphangiogenic disease (8). Many scholars have argued that it is due to congenital developmental deformity, leading to abnormal hyperplasia or dilatation of the lymphatic channels (8). Moreover, Allen et al. reported that it is related to some acquired factors, such as repeated infection, surgery, trauma, and radiotherapy, causing lymphatic fluid reflux disorders (9). Histologically, LA is usually divided into lymphangioma (simplex), cavernous lymphangioma, and CL. Clinically, CL is the most common type (1), mostly developing in the neck (75%), followed by the axilla (20%), and rarely (<1%) in the abdomen, which is common in children but rare in adults (10–12). Due to the slow growth, low tension, and certain degree of plasticity of the CL, there are generally no evident symptoms in the early stage, and the later symptoms depend on the degree of cyst expansion and the extrusion of surrounding tissues. Local masses are usually used during clinic visits. Preoperative diagnosis is mainly based on imaging examination, and diagnosis depends on pathological examination and/or immunohistochemical D2-40 expression (4). The mainstay of treatment for CL is surgical excision. Due to its characteristic of spreading and infiltrating growth along the tissue space, only 18–50% of patients undergo complete resection (13). However, even after complete resection, there is still a recurrence rate of 0–27% (14).

In this case, the lesion was confined to the right inguinal region in the early stage and was a multilocular cystic mass, with thin and smooth cyst walls and septum, and the cyst cavity was filled with lymphs. The longitudinal axis showed a baggy change, showing “creeping growth” without enhancement on contrast-enhanced scan (15). MRI examination in 2016 revealed that the scope of the lesion was significantly expanded, the signal intensity was uneven, and the septum was thickened. In 2018, the number of the fourth recurrent lesion increased significantly, the scope was larger, the solid components in the capsule increased, and the thickness of the septum was uneven or damaged and blurred. After 16 years of follow-up, we found that the recurrence time interval of the lesions decreased, the growth rate became significantly faster, the scope of invasion was larger, and the signal intensity changed from uniform to uneven. Moreover, regarding the appearance of definite solid components, the septum changed from thin to uneven and then to blurring. Imaging completely revealed the process of malignant CL transforming into LAS. The pathological section from 2008 to 2016 revealed that the lesion evolved from benign to malignant, mainly manifested as endothelial cells from loose to dense, the degree of atypia gradually increased, and pathological mitosis occurred. Due to poor physical condition, the patient did not receive further treatment after being diagnosed with LAS. The patient’s inguinal mass has been treated for several years, and only the fourth recurrence was accompanied by abdominal distension, which was considered to be caused by tumor compression. The related tumor index did not increase, and no evidence of metastasis was found in the other organs. The patient eventually died of complications caused abdominal organ compression 2 years after being diagnosed with LAS.

LAS is a rare invasive tumor arising from lymphatic endothelial cells, with a high degree of malignancy and poor outcomes. Its survival rate is only 15 to 30 months (16), almost all of which are secondary to chronic lymphedema caused by various causes (mainly after surgery or radiotherapy) (2, 3). Typical LAS cases occur in patients with chronic lymphedema following breast cancer, with the skin of the head and neck being the most common site of origin (2). In our case, there was no lymphedema before malignant transformation, and abdominal wall edema was only observed in the last imaging examination (2018). However, no known background that might induce LAS was found. Some cases of benign tumors progressing to malignant tumors have been confirmed, such as neurofibroma developing into malignant schwannoma (17) and hemangioma malignant transforming into angiosarcoma (18). Although no case of malignant transformation of CL to LAS has been reported, we hypothesized that such a possibility exists through the clinical, imaging, and pathological evolution of this case, and multiple recurrence and surgical stimulation are possibly the precipitating factors of CL malignant transformation.

There are few reports about LAS, and the relevant clinical treatment options are still being explored. Cook MR et al. (19) reported a dog that underwent amputation after the diagnosis of LAS on the left elbow was confirmed and doxorubicin chemotherapy was administered, but the tumor still recurred at the amputation site 7 months later. A 31-year-old female patient with LAS of the right hip was treated with docetaxel, and although the lymphatic leakage had initially improved, the tumor did not shrink and the patient eventually died of complications a year and a half later (3). In this case, the targeted therapy with apatinib had a certain curative effect at the initial stage, but the long-term effect is still uncertain. The effect of a single treatment regimen may be limited, and a better therapeutic effect may be achieved by multimodal treatment such as radiotherapy and chemotherapy after operation. We found that a study (20) made a similar point that among the 12 dogs with LAS, those who received exclusive surgical excision, or a combination of surgery, radiation and/or chemotherapy had the longest survival times.

Here, we report a rare case of adult inguinal CL developing into LAS. For CL, imaging examination is a significantly important means of diagnosis, clean surgical resection is the main treatment option, and regular follow-up is necessary. For recurrent lesions, if the growth rate is accelerated, the recurrence interval is shortened, realistic components appear in the cyst, and the septum is uneven and thickened. Thus, high vigilance should be taken for CL’s malignant transformation into LAS.

## Data Availability Statement

The original contributions presented in the study are included in the article/supplementary material. Further inquiries can be directed to the corresponding author.

## Ethics Statement

Ethical review and approval was not required for the study on human participants in accordance with the local legislation and institutional requirements. The patients/participants provided their written informed consent to participate in this study. Written informed consent was obtained from the individual(s) for the publication of any potentially identifiable images or data included in this article.

## Author Contributions

XX: designed the study. HY and JL: collected and analyzed the data. HY, QM, and LZ: manuscript writing. XX: reviewed and edited the manuscript. All authors contributed to the article and approved the submitted version.

## Funding

This research was funded by Scientific Research Project of Hubei Provincial Health Commission (WJ2021F014) and Wuhan Municipal Health Commission (WX20Z46).

## Conflict of Interest

The authors declare that the research was conducted in the absence of any commercial or financial relationships that could be construed as a potential conflict of interest.

## Publisher’s Note

All claims expressed in this article are solely those of the authors and do not necessarily represent those of their affiliated organizations, or those of the publisher, the editors and the reviewers. Any product that may be evaluated in this article, or claim that may be made by its manufacturer, is not guaranteed or endorsed by the publisher.
